# Diethyl 6*H*,12*H*-5,11-methano­dibenzo[*b*,*f*][1,5]diazo­cine-1,7-dicarboxyl­ate

**DOI:** 10.1107/S1600536808042967

**Published:** 2008-12-20

**Authors:** M. Delower H. Bhuiyan, Jack K. Clegg, Andrew C. Try

**Affiliations:** a Department of Chemistry and Biomolecular Sciences, Building F7B, Macquarie University, NSW 2109, Australia; b Crystal Structure Analysis Facility, School of Chemistry, F11, The University of Sydney, NSW 2006, Australia

## Abstract

In the mol­ecule of the title compound, C_21_H_22_N_2_O_4_, the 1,7-diethyl ester analogue of Tröger’s base, the dihedral angle between the two benzene rings is 93.16 (3)°; the mol­ecule is *C*_2_ symmetric.

## Related literature

For background to the synthesis of Tröger’s base products, see: Hansson *et al.* (2003 ▶); Solano *et al.* (2005 ▶); Bhuiyan *et al.* (2007 ▶); Didier & Sergeyev (2007 ▶); Zhu *et al.* (2008 ▶); Vande Velde *et al.* (2008 ▶). For related structures, see: Faroughi *et al.* (2006 ▶); Bhuiyan *et al.* (2006 ▶).
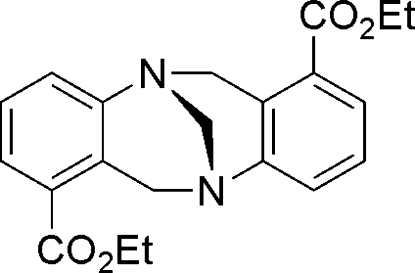


## Experimental

### 

#### Crystal data

C_21_H_22_N_2_O_4_
*M* *_r_* = 366.41
Monoclinic, 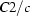

*a* = 14.306 (3) Å
*b* = 9.251 (2) Å
*c* = 15.081 (4) Å
β = 118.149 (4)°
*V* = 1759.8 (7) Å^3^
*Z* = 4
Mo *K*α radiation
μ = 0.10 mm^−1^
*T* = 150 (2) K
0.47 × 0.30 × 0.19 mm

#### Data collection

Bruker SMART 1000 CCD diffractometer
Absorption correction: multi-scan (*SADABS*; Sheldrick, 1996 ▶) *T*_min_ = 0.856, *T*_max_ = 0.980
8475 measured reflections
2135 independent reflections
1925 reflections with *I* > 2σ(*I*)
*R*_int_ = 0.020

#### Refinement

*R*[*F* ^2^ > 2σ(*F* ^2^)] = 0.036
*wR*(*F* ^2^) = 0.099
*S* = 1.04
2135 reflections
124 parameters
H-atom parameters constrained
Δρ_max_ = 0.33 e Å^−3^
Δρ_min_ = −0.19 e Å^−3^

### 

Data collection: *SMART* (Siemens, 1995 ▶); cell refinement: *SAINT* (Siemens, 1995 ▶); data reduction: *SAINT* and *XPREP* (Siemens, 1995 ▶); program(s) used to solve structure: *SIR97* (Altomare *et al*. 1999 ▶); program(s) used to refine structure: *SHELXL97* (Sheldrick, 2008 ▶); molecular graphics: *ORTEP-3* (Farrugia, 1997 ▶) and *WinGX32* (Farrugia, 1999 ▶); software used to prepare material for publication: *enCIFer* (Allen *et al.*, 2004 ▶).

## Supplementary Material

Crystal structure: contains datablocks global, I. DOI: 10.1107/S1600536808042967/zl2169sup1.cif

Structure factors: contains datablocks I. DOI: 10.1107/S1600536808042967/zl2169Isup2.hkl

Additional supplementary materials:  crystallographic information; 3D view; checkCIF report

## Figures and Tables

**Fig. 1.**
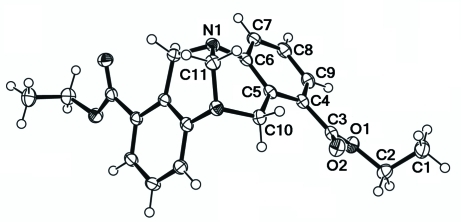
View of the title compound, showing the atomic numbering scheme. Displacement ellipsoids are shown at the 50% probability level. Symmetry code used to generate equivalent atoms: 2-x, y, 1.5-z.

**Fig. 2.**
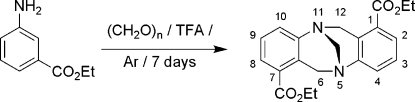
Synthetic scheme for the synthesis of the title compound showing the numbering system used in naming the compound.

## supplementary crystallographic information

### Comment

Dibenzo Tröger's base analogues are formed from the acid catalysed condensation of an aniline with either formaldehyde or formaldehyde equivalents. It was a long-held belief that a *para*-substituent was required on the aniline to prevent polymerization during the Tröger's base reaction and that the presence of an electron-withdrawing group would result in neglible yields of Tröger's base products. These beliefs have been proved to be incorrect, with the synthesis of tetranitro- (Bhuiyan *et al.*, 2007) and octafluoro- (Vande Velde *et al.*, 2008) analogues (in yields of 11% and 37%, respectively), and the synthesis of Tröger's base analogues from 2- and 3-substituted anilines lacking a substitutent in the *para*-position (Hansson *et al.*, 2003), and even from aniline itself (Didier & Sergeyev, 2007). The title compound is another example of a Tröger's base analogue unsubstituted in the 2,8-positions. An important feature of all Tröger's base analogues is the V-shaped structure of the compounds. The dihedral angle between the aromatic rings has been measured for over 25 simple dibenzo Tröger's base analogues and has been found to lie between 82° (Solano *et al.*, 2005) and 110° (Zhu *et al.*, 2008). The X-ray structures of two related Tröger's base esters have also been reported (Faroughi *et al.*, 2006; Bhuiyan *et al.*, 2006). It is noteworthy that the title compound was the sole Tröger's base analogue isolated from the reaction and results from carbon-carbon bond formation at the more hindered *ortho*-site, relative to the aniline amino group.

The title compound, Fig. 1, crystallizes in space group *C*2/*c* and it was prepared as outlined in Fig. 2.

### Experimental

Ethyl 3-aminobenzoate (2.0 g, 12.1 mmol) and paraformaldehyde (582 mg, 19.38 mmol) were dissolved in trifluoroacetic acid (75 ml) and the mixture was stirred under an argon atmosphere in the dark 7 days. The reaction mixture was then basified with a solution of concentrated ammonia (80 ml) in water (120 ml). A saturated sodium hydrogen carbonate solution (100 ml) was added and the crude material was extracted into ethyl acetate (3 × 75 ml). The combined organic layers were washed with brine (100 ml), dried over anhydrous sodium sulfate, filtered and evaporated to dryness to yield an orange solid. The crude material was purified by recrystallization from hexane to afford the title compound (760 mg, 34%) as a white solid and a racemic mixture, m.p. 441–443 K.

Single crystals of the title compound were produced by slow evaporation of a dichloromethane solution.

### Refinement

C-bound H atoms were included in idealized positions and refined using a riding model. Methylene, aromatic and methyl C—H bond lengths were fixed at 0.99, 0.95 and 0.98 Å, respectively. *U*_iso_(H) values were fixed at 1.2*U*_eq_(C) for methylene and aromatic H atoms, and at 1.5*U*_eq_(C) for methyl H atoms.

### Crystal data

**Table:**

C_21_H_22_N_2_O_4_	*F*(000) = 776
*M**_r_* = 366.41	*D*_x_ = 1.383 Mg m^−^^3^
Monoclinic, *C*2/*c*	Melting point: 441 K
Hall symbol: -C 2yc	Mo *K*α radiation, λ = 0.71073 Å
*a* = 14.306 (3) Å	Cell parameters from 5209 reflections
*b* = 9.251 (2) Å	θ = 2.7–28.5°
*c* = 15.081 (4) Å	µ = 0.10 mm^−^^1^
β = 118.149 (4)°	*T* = 150 K
*V* = 1759.8 (7) Å^3^	Shard, colourless
*Z* = 4	0.47 × 0.30 × 0.19 mm

### Data collection

**Table:**

Bruker SMART 1000 CCD diffractometer	2135 independent reflections
Radiation source: sealed tube	1925 reflections with *I* > 2σ(*I*)
graphite	*R*_int_ = 0.020
ω scans	θ_max_ = 28.5°, θ_min_ = 2.7°
Absorption correction: multi-scan (*SADABS*; Sheldrick, 1996)	*h* = −19→19
*T*_min_ = 0.856, *T*_max_ = 0.980	*k* = −12→11
8475 measured reflections	*l* = −20→20

### Refinement

**Table:**

Refinement on *F*^2^	Primary atom site location: structure-invariant direct methods
Least-squares matrix: full	Secondary atom site location: difference Fourier map
*R*[*F*^2^ > 2σ(*F*^2^)] = 0.036	Hydrogen site location: inferred from neighbouring sites
*wR*(*F*^2^) = 0.099	H-atom parameters constrained
*S* = 1.04	*w* = 1/[σ^2^(*F*_o_^2^) + (0.0513*P*)^2^ + 1.146*P*] where *P* = (*F*_o_^2^ + 2*F*_c_^2^)/3
2135 reflections	(Δ/σ)_max_ = 0.001
124 parameters	Δρ_max_ = 0.33 e Å^−^^3^
0 restraints	Δρ_min_ = −0.18 e Å^−^^3^

### Special details

**Table:**

Experimental. The crystal was coated in Exxon Paratone N hydrocarbon oil and mounted on a thin mohair fibre attached to a copper pin. Upon mounting on the diffractometer, the crystal was quenched to 150(K) under a cold nitrogen gas stream supplied by an Oxford Cryosystems Cryostream and data were collected at this temperature.
Geometry. All e.s.d.'s (except the e.s.d. in the dihedral angle between two l.s. planes) are estimated using the full covariance matrix. The cell e.s.d.'s are taken into account individually in the estimation of e.s.d.'s in distances, angles and torsion angles; correlations between e.s.d.'s in cell parameters are only used when they are defined by crystal symmetry. An approximate (isotropic) treatment of cell e.s.d.'s is used for estimating e.s.d.'s involving l.s. planes.
Refinement. Refinement of *F*^2^ against ALL reflections. The weighted *R*-factor *wR* and goodness of fit *S* are based on *F*^2^, conventional *R*-factors *R* are based on *F*, with *F* set to zero for negative *F*^2^. The threshold expression of *F*^2^ > σ(*F*^2^) is used only for calculating *R*-factors(gt) *etc*. and is not relevant to the choice of reflections for refinement. *R*-factors based on *F*^2^ are statistically about twice as large as those based on *F*, and *R*- factors based on ALL data will be even larger.

### Fractional atomic coordinates and isotropic or equivalent isotropic displacement parameters (Å^2^)

**Table:**

	*x*	*y*	*z*	*U*_iso_*/*U*_eq_	Occ. (<1)
C1	0.83967 (10)	0.37215 (14)	1.11492 (10)	0.0346 (3)	
H1A	0.7669	0.4024	1.0692	0.052*	
H1B	0.8563	0.3944	1.1844	0.052*	
H1C	0.8465	0.2679	1.1081	0.052*	
C2	0.91539 (9)	0.45165 (12)	1.08889 (8)	0.0291 (2)	
H2A	0.9125	0.5564	1.1007	0.035*	
H2B	0.9886	0.4177	1.1328	0.035*	
C3	0.92507 (8)	0.30443 (11)	0.96454 (8)	0.0217 (2)	
C4	0.88028 (8)	0.27735 (11)	0.85453 (8)	0.0205 (2)	
C5	0.92893 (8)	0.17644 (10)	0.81900 (8)	0.0194 (2)	
C6	0.87857 (8)	0.14597 (11)	0.71558 (8)	0.0201 (2)	
C7	0.78414 (8)	0.21655 (12)	0.65048 (8)	0.0238 (2)	
H7	0.7503	0.1941	0.5808	0.029*	
C8	0.73945 (8)	0.31834 (12)	0.68617 (8)	0.0262 (2)	
H8	0.6764	0.3673	0.6411	0.031*	
C9	0.78727 (8)	0.34861 (12)	0.78832 (8)	0.0242 (2)	
H9	0.7566	0.4180	0.8133	0.029*	
C10	1.03523 (8)	0.10512 (11)	0.88716 (8)	0.0211 (2)	
H10A	1.0846	0.1783	0.9335	0.025*	
H10B	1.0250	0.0294	0.9282	0.025*	
C11	1.0000	−0.05009 (15)	0.7500	0.0236 (3)	
H11A	0.9674	−0.1129	0.7810	0.028*	0.50
H11B	1.0326	−0.1129	0.7190	0.028*	0.50
N1	0.91816 (7)	0.03970 (9)	0.67236 (6)	0.0214 (2)	
O1	0.88792 (6)	0.42718 (8)	0.98415 (6)	0.02669 (19)	
O2	0.98621 (6)	0.22448 (9)	1.02960 (6)	0.0285 (2)	

### Atomic displacement parameters (Å^2^)

**Table:**

	*U*^11^	*U*^22^	*U*^33^	*U*^12^	*U*^13^	*U*^23^
C1	0.0422 (7)	0.0347 (6)	0.0350 (6)	−0.0024 (5)	0.0249 (5)	−0.0045 (5)
C2	0.0339 (6)	0.0266 (5)	0.0284 (5)	−0.0023 (4)	0.0161 (5)	−0.0066 (4)
C3	0.0205 (5)	0.0209 (5)	0.0272 (5)	−0.0007 (4)	0.0142 (4)	0.0015 (4)
C4	0.0195 (5)	0.0198 (5)	0.0247 (5)	−0.0003 (4)	0.0124 (4)	0.0025 (4)
C5	0.0178 (4)	0.0174 (4)	0.0246 (5)	−0.0002 (3)	0.0113 (4)	0.0032 (4)
C6	0.0186 (4)	0.0182 (4)	0.0256 (5)	−0.0032 (4)	0.0121 (4)	0.0005 (4)
C7	0.0192 (5)	0.0274 (5)	0.0234 (5)	−0.0028 (4)	0.0090 (4)	0.0014 (4)
C8	0.0182 (5)	0.0294 (5)	0.0293 (5)	0.0037 (4)	0.0097 (4)	0.0062 (4)
C9	0.0210 (5)	0.0240 (5)	0.0305 (5)	0.0039 (4)	0.0146 (4)	0.0033 (4)
C10	0.0208 (5)	0.0206 (5)	0.0230 (5)	0.0031 (4)	0.0113 (4)	0.0027 (4)
C11	0.0254 (7)	0.0177 (6)	0.0296 (7)	0.000	0.0145 (6)	0.000
N1	0.0215 (4)	0.0191 (4)	0.0257 (4)	−0.0021 (3)	0.0128 (4)	−0.0009 (3)
O1	0.0322 (4)	0.0230 (4)	0.0277 (4)	0.0043 (3)	0.0165 (3)	0.0007 (3)
O2	0.0307 (4)	0.0292 (4)	0.0258 (4)	0.0078 (3)	0.0135 (3)	0.0048 (3)

### Geometric parameters (Å, °)

**Table:**

C1—C2	1.5065 (17)	C6—C7	1.4014 (14)
C1—H1A	0.9800	C6—N1	1.4354 (13)
C1—H1B	0.9800	C7—C8	1.3820 (16)
C1—H1C	0.9800	C7—H7	0.9500
C2—O1	1.4544 (13)	C8—C9	1.3876 (16)
C2—H2A	0.9900	C8—H8	0.9500
C2—H2B	0.9900	C9—H9	0.9500
C3—O2	1.2103 (13)	C10—N1^i^	1.4770 (13)
C3—O1	1.3445 (13)	C10—H10A	0.9900
C3—C4	1.4909 (15)	C10—H10B	0.9900
C4—C9	1.3959 (14)	C11—N1	1.4623 (12)
C4—C5	1.4124 (14)	C11—H11A	0.9900
C5—C6	1.4039 (15)	C11—H11B	0.9900
C5—C10	1.5262 (13)		
			
C2—C1—H1A	109.5	C8—C7—H7	119.5
C2—C1—H1B	109.5	C6—C7—H7	119.5
H1A—C1—H1B	109.5	C7—C8—C9	119.58 (10)
C2—C1—H1C	109.5	C7—C8—H8	120.2
H1A—C1—H1C	109.5	C9—C8—H8	120.2
H1B—C1—H1C	109.5	C8—C9—C4	120.20 (10)
O1—C2—C1	110.36 (9)	C8—C9—H9	119.9
O1—C2—H2A	109.6	C4—C9—H9	119.9
C1—C2—H2A	109.6	N1^i^—C10—C5	111.09 (8)
O1—C2—H2B	109.6	N1^i^—C10—H10A	109.4
C1—C2—H2B	109.6	C5—C10—H10A	109.4
H2A—C2—H2B	108.1	N1^i^—C10—H10B	109.4
O2—C3—O1	123.18 (10)	C5—C10—H10B	109.4
O2—C3—C4	124.49 (10)	H10A—C10—H10B	108.0
O1—C3—C4	112.31 (9)	N1^i^—C11—N1	110.77 (11)
C9—C4—C5	120.99 (10)	N1^i^—C11—H11A	109.5
C9—C4—C3	118.74 (9)	N1—C11—H11A	109.5
C5—C4—C3	120.21 (9)	N1^i^—C11—H11B	109.5
C6—C5—C4	117.88 (9)	N1—C11—H11B	109.5
C6—C5—C10	119.18 (9)	H11A—C11—H11B	108.1
C4—C5—C10	122.88 (9)	C6—N1—C11	111.33 (8)
C7—C6—C5	120.31 (9)	C6—N1—C10^i^	112.54 (8)
C7—C6—N1	117.22 (9)	C11—N1—C10^i^	107.39 (7)
C5—C6—N1	122.43 (9)	C3—O1—C2	115.98 (8)
C8—C7—C6	120.98 (10)		
			
O2—C3—C4—C9	159.42 (10)	C7—C8—C9—C4	0.40 (16)
O1—C3—C4—C9	−19.11 (13)	C5—C4—C9—C8	1.66 (16)
O2—C3—C4—C5	−17.78 (16)	C3—C4—C9—C8	−175.52 (10)
O1—C3—C4—C5	163.70 (9)	C6—C5—C10—N1^i^	13.93 (12)
C9—C4—C5—C6	−2.43 (14)	C4—C5—C10—N1^i^	−163.33 (9)
C3—C4—C5—C6	174.70 (9)	C7—C6—N1—C11	−165.60 (8)
C9—C4—C5—C10	174.85 (9)	C5—C6—N1—C11	12.31 (12)
C3—C4—C5—C10	−8.01 (14)	C7—C6—N1—C10^i^	73.76 (11)
C4—C5—C6—C7	1.21 (14)	C5—C6—N1—C10^i^	−108.32 (10)
C10—C5—C6—C7	−176.18 (9)	N1^i^—C11—N1—C6	−51.41 (6)
C4—C5—C6—N1	−176.64 (8)	N1^i^—C11—N1—C10^i^	72.21 (6)
C10—C5—C6—N1	5.97 (14)	O2—C3—O1—C2	−7.03 (15)
C5—C6—C7—C8	0.80 (15)	C4—C3—O1—C2	171.51 (8)
N1—C6—C7—C8	178.76 (9)	C1—C2—O1—C3	−83.19 (12)
C6—C7—C8—C9	−1.62 (16)		

Symmetry codes: (i) −*x*+2, *y*, −*z*+3/2.
